# Deconstructing Immersion in the Experience Economy Framework for Immersive Dining Experiences through Mixed Reality

**DOI:** 10.3390/foods11233780

**Published:** 2022-11-23

**Authors:** Dai-In Danny Han, Malu Boerwinkel, Mata Haggis-Burridge, Frans Melissen

**Affiliations:** 1 Academy for Hotel and Facility, Breda University of Applied Sciences, Mgr. Hopmansstraat 2, 4817 JS Breda, The Netherlands; 2 Research Centre Future of Food, Zuyd University of Applied Sciences, Bethlehemweg 2, 6222 BM Maastricht, The Netherlands; 3 Academy for Games, and Media, Breda University of Applied Sciences, Mgr. Hopmansstraat 1, 4817 JT Breda, The Netherlands

**Keywords:** immersive experience, retrospective think aloud protocol, galvanic skin response, mixed reality

## Abstract

In this study, we test the immersive character in an interactive content narrative developed for Microsoft HoloLens 2 mixed reality glasses in the dining context. We use retrospective think aloud protocol (RTAP) and galvanic skin response (GSR) to explore different types of immersion that can be created through interactive content narratives. Leaning on the core dimensions of the experience economy, we expand the current understanding on the role of immersion through integration of four immersive experience facilitators. The study revealed that these immersive experience facilitators occur simultaneously and can be enhanced through interactive content narrative design. Perceived novelty and curiosity were identified as key determinants to keep consumers engaged in the immersive experience and engage with the content. The study verifies the use of galvanic skin response in combination with retrospective think aloud protocol as a suitable approach to measure emotional engagement potential in interpreting consumers’ recollection of immersive experiences.

## 1. Introduction

The concept of immersion within the hospitality industry has been used in various settings, such as in studies investigating hospitality experiences [1] or the state of mind of guests [2]. In times when the consequences of the COVID-19 pandemic have yet to show its full impact on the food and wider hospitality industry, it is increasingly clear that our industry will need to redefine itself to become more resilient and sustainable in the post-COVID-19 era. The pandemic has painfully uncovered the limited global access to nutritious food as aspired in the Sustainable Development Goals and exposed challenges in our food system from food production to diminishing consumer experiences [3]. The forced shut down of restaurants and food outlets across the globe has created a gap in food consumption and experience patterns for consumers and businesses in the food service sector. Using immersive experiences could be a way to stimulate and revive innovative initiatives and adapt consumer behavior toward more sustainable food choices, healthy behavior, spark curiosity to new forms and approaches to food. A few recent studies indicated that immersion affected post-experience behavior such as intention to revisit or customer loyalty [4,5]. Immersive experiences also offer a promising approach to employing evolutionary tendencies [6] by bringing abstract concepts into the immediate (mental) environment of consumers. Through the use of immersive technologies, consumers could potentially be stimulated to sense sustainability issues (e.g., climate change) that could not be grasped otherwise. While the extant literature is available on studies to stimulate consumer attitude and behavior through storytelling [7], our knowledge on how to employ interactive content narratives in mixed reality is limited. 

In the food and dining context, the concept of immersion has played a key role in the enjoyment of food consumption. For instance, the role of spatial immersion was explored in an Eat & Travel concept, which created a dining experience while virtually traveling to the destination where the food originated [8]. Emerging technologies such as augmented reality (AR) and virtual reality (VR) have further fueled the dimension of spatial immersion [9]. One of the most common approaches is the use of projection mapping such as “Le Petit Chef” developed by Skullmapping, which augments engaging stories of a miniature character on the table of guests, offering an entertainment value while waiting for meals and drinks to be prepared (for reference see www.skullmapping.com, accessed on 7 November 2022). This has resulted in changing consumer expectations in the seamless implementation of technology and resulting immersive experience. Studies have shown that the enjoyment of food in the dining experience is influenced by more than the food quality alone, for instance by recognizing the influence of the social environment. It was concluded that dining experiences are evaluated in consideration of the overall enjoyment and evaluation of external components generating the complete experience [10,11]. In the museum context, the social context in creating interactive experiences was revealed to be crucial [12]. However, social immersion as a distinct form of immersion in the food context has been limitedly explored to date, providing us with little knowledge on how external factors like the social environment can be stimulated and better controlled through immersion. Immersion was also studied as a form of an enhancement of taste perception using electrical pulses and virtual overlays to modify the taste experience [13]. It was revealed that technological stimuli could alter taste perceptions and create immersive taste experiences. However, as in many other studies, other forms of immersion (e.g., spatial immersion) have not been included in their study, limiting the understanding of the potential types and degrees of immersion that can influence the food experience. Overall, consumer experience literature in the food and dining context studying immersive experiences (as an isolated construct) is scarce and does not provide much empirical evidence on design criteria and effects of such experiences. Use cases have been limited, as ways to generate immersive experiences are not well understood in consumer experience literature. For instance, the effect of matching and mismatching immersive environments to a food item was investigated in a prior study concluding that immersive environments generated through immersive technologies (e.g., immersive cave setting) had high potential to recreate real scenarios for consumer studies on food product testing [14]. A systematic literature review revealed that immersive environments were particularly interesting for sensory research settings, which aim for high external validity [15]. While prior studies in this field claim to use immersive environments and contexts, they do not address immersive experiences as a mechanism to drive consumer attitude and behavior. Prior studies specifically call for further research to identify factors that contribute most to the immersive experience [14]. To date, immersive experiences are left as a desired state rather than an intentionally designed phenomenon which greatly limits our ability to control and manage consumer experiences and reap the benefits (e.g., full consumer attention and post-experience attitudes) that immersive consumer experiences could provide. To the best of our knowledge, there is currently no study that investigates the effect of purposefully designed facilitators for creating immersive experiences through interactive content narratives in mixed reality. It begs two questions, first, how content narratives in immersive technologies can be better designed to create intentional immersive experiences. Second, how consumers’ perceived immersive experience effect develops over time, which this study aims to address.

The aims of this study are two-fold. First, this study tests the immersive experience of an interactive content narrative developed for Microsoft HoloLens 2 mixed reality glasses to extend our understanding of the consumer experience in this context learning on the experience economy framework [16]. While the components of the experience economy framework have been studied in multiple contexts, we remain to have little understanding on the role of immersion in designing and measuring consumer experiences. With this study, we aim to expand our current understanding of the experience economy framework by investigating immersion as a key construct. The prototype used in this study was developed based on four distinct immersive experience facilitators [17]. In our study, we seek consumer insights while engaging with the interactive MR content to test the effectiveness of immersive experience facilitators during the experience. Thus, second, this study aims to explore how immersive experiences develop over the duration of the consumer engagement with the MR content. 

## 2. Literature Review

### 2.1. Experience Economy Framework

**Figure 1:**
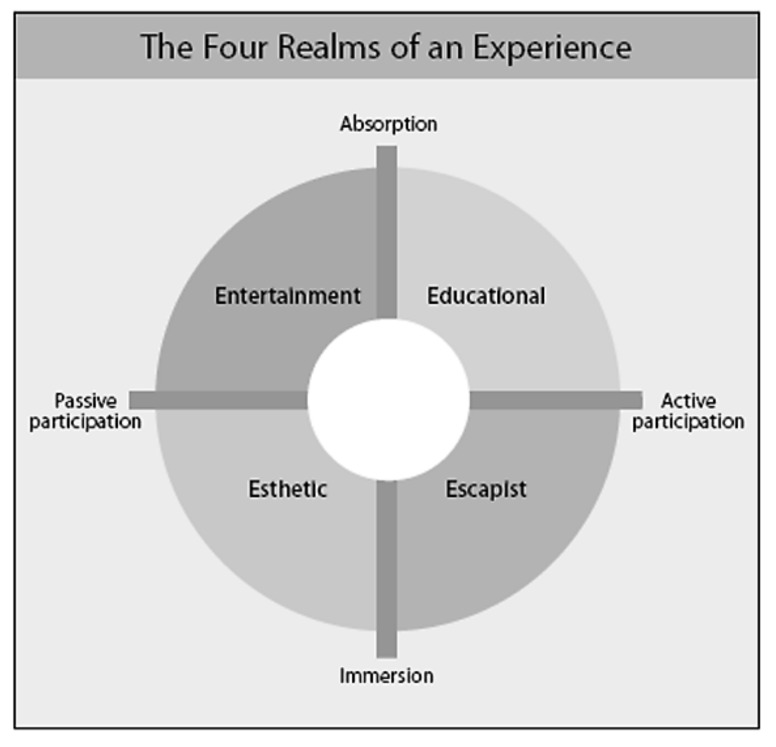
Four realms of the experience economy adopted from [16]. Reprinted with permission from ‘Welcome to the Experience Economy’ by B. Joseph Pine II; James H. Gilmore. Harvard Business Review, July 1998. Copyright 1998 by Harvard Business Publishing; all rights reserved.

With the experience economy shifting the paradigm of economic offerings from businesses merely performing a function to businesses providing an experience, companies must learn to effectively stage, sell, and market engaging experiences to gain competitive advantage [16]. The model of the experience economy has been recognized and integrated into various industries such as the retail industry [18,19], architecture and urban planning [20,21,22], and culture and education [23]. However, the focus on creating memorable experiences seems to be most prevalent in the tourism and hospitality industry [24,25], where many service providers seek to satisfy the modern consumer’s need to find the optimal memorable experience, beyond simply consuming the function of products and services [16,26]. Four categories of experiences were recognized, divided by the dimensions of passive to active customer participation and customer absorption to customer immersion (Figure 1) [16]. Active participation includes customers directly influencing the experience while immersion describes being physically or virtually enveloped by an experience [27]. Absorption further involves the engagement of the consumer’s mind and attention. According to Pine and Gilmore “the richest experiences encompass aspects of all four realms, forming a “sweet spot” around the area where the spectra meet” [16] (p. 102).

A number of prior studies investigated the role of immersive technologies on the realms of the experience economy. AR was argued to be an effective tool to generate educational experiences through the capability to offer interactive content in the immediate surrounding of learners [28]. It was also revealed that immersive technologies offered experiences with heightened levels of enjoyment; however, concluding that this often resulted in entertainment experiences, which are more passively consumed as opposed to the interactive value offered by immersive technologies [4]. They argued that immersion through escapism and esthetics in current and future AR applications will become increasingly important to engage consumers in fulfilling their experience needs. While the framework of the experience economy was praised for its flexibility and overall conceptualization, concerns remain on the measurability of components and lack of further insight into how these elements are designed or staged. While the realms of the experience economy have been investigated in prior studies in the context of immersive technologies or food tourism, the role of immersion and how immersive experiences can be generated remains unclear. Therefore, this study aims to extend prior research on the experience economy framework by contributing to the understanding on the role of immersion and how immersive experiences in the food and dining context can be generated for consumers. The following chapter provides an analysis of immersive experiences in the food and dining context and how they relate to the experience economy framework. 

### 2.2. Immersive Experiences in Food and Dining 

In the food and dining context, immersion has largely been studied in relation to evaluating guest experiences. In restaurants such as the Fat Duck, Heston Blumenthal experimented with adding sound through an iPod that would allow guests to listen to waves at the sea while having Oysters. The Michelin star chef suggested that such sensory additions would not only create immersive experiences, but also stimulate taste buds making the Oysters taste “stronger and saltier” [29]. Previous literature on immersion in the food and dining context indicates that various types of immersion can be attributed to purpose-driven outcomes. For instance, systems immersion [30,31] suggests increasing learning and understanding, while empathic [30] and social immersion [8] was found to trigger emotional responses in study participants. It thus seems that immersion can be achieved for educational and entertainment purposes as well, and is not reserved for creating esthetic and escapist experiences as illustrated in the experience economy framework [16]. Prior studies demonstrated that educational experiences can embody immersive characters which can enhance learning experiences [30,31]. Spatial immersion was revealed to be the most prominent form of immersion in the food and dining context and all studies confirmed that spatial immersion increased cognitive engagement during the experience consumption. Spatial immersion is arguably closely related to the sense of escapism as the experiencer is mentally and emotionally engaged in an immersive experience. Considering that spatial immersion is achieved through consistent design of environmental artifacts, it closely aligns with the experience economy framework. In contrast to the present approaches in the use of immersion to generate experience, prior studies used virtual augmentations to modify taste sensations and increase perceived immersion as a taste experience [13]. Their study aimed to increase perceptual immersion by modifying taste buds using electrical pulses on the tip of the tongue and virtual overlays to modify colors of products. While their approach is arguably linked to systems immersion, it does not follow the commonly used approach to systems immersion as used in other studies presented in this section. Instead, prior approaches appear to relate to manipulating esthetic components of the experience [13], while active participation is required to engage in the experience itself. It is thus not in line with the experience economy framework, which suggests that the framework offers a generalization of experiences and do not fully capture different types of immersive experiences that can be created. This indicates that the definition and approach to various types of immersion are conceptually still limitedly explored, highlighting the need to conceptualize immersion in the food and dining context to advance and exploit the opportunities of immersive experiences in our industry. 

Immersive technologies such as VR and CAVE settings through projection mapping provide the opportunity to enhance immersive settings in the food and dining context. A study investigating virtual travel and dining would allow guests to be immersed in a virtual CAVE setting to virtually travel to another destination while enjoying related food [8]. While testing their Eat & Travel restaurant, it was revealed that spatial immersion through a technology-mediated CAVE setting increased guest enjoyment of the dining experience and desire to learn and share their experience with others. This approach is in line with the experience economy framework, suggesting the use of immersive technology to facilitate escapism through spatial immersion. Interestingly, in their study, a combination of various types of immersion was used next to spatial immersion. Social immersion was implemented in the form of a virtual cruise captain that would introduce guests to the sights appearing in the environment. The virtual travel experience covered various sights which are relatable to narrative immersion. It appears to suggest that entertainment components that are passively enjoyed can further enhance the immersive experience through spatial immersion. Another study experimented with spatial immersion to create various bar atmospheres and investigate whether stimulating changes in the environment through visual projections would influence declarative drink choices [2]. Their study found that spatial immersion through visual projections could be used to further explore the influence of contextual variables of food and drink choices. In this context, social immersion could play a role by stimulating the sense of place and connecting them to socially appropriate selection of drinks. However, although the experiment in this study was set up for participants to join the research in groups to replicate a realistic bar scene, the influence of social immersion was not measured. Similar to the Eat & Travel restaurant [8], it seems that multiple realms of the experience economy can be employed simultaneously to reach an enhanced immersive experience. Similar CAVE setups have been developed in other studies [32] revealing similar results. In their immersive CAVE setting to provide multisensory gastronomic experiences, they used visual, auditory, olfactory, and kinesthetic augmentations via projections, LED lighting, smoke machines and scent air machines to create an immersive space. The multi-sensory environment aimed to seamlessly engage the senses of restaurant guests resulting in an immersive experience with limited contribution needed from the guest. Instead, wireless motion tracking controllers allowed staff to interact with the virtual augmentations in order to create flexible interactive experiences to suit different types of guests and moods. Interactive elements can be incorporated to increase guest interaction and information provision in the virtual environment. This was further expanded on studies of spatially immersive environments and used an augmented virtuality setup in their study to develop immersive gastronomic experiences that would virtually transport participants into different environments while using mixed reality head-sets to consume actual food items [1]. This approach allowed for pairing food items with the virtual environment through 360 degree videos to spatially immerse participants into the food consumption. It was argued that sufficient levels of immersion and satisfaction could be generated through cost-efficient and widely accessible smartphone-based virtual environments. This finding does not only demonstrate the possibility to generate spatial immersion through VR headsets to create flexible virtual research environments, but also highlights the potential of creating escapist experiences through spatial immersion in VR in combination with active participation following the logic of the experience economy framework. 

The role of immersion was studied as an approach to teach food science and revealed that students could recall basic food concepts of food science more prominently through an immersive learning experience rather than through provided information in a passive learning setup [31]. As a team-based learning environment using a laboratory setting to teach food science concepts, the study reflects the use of systems immersion in the hands-on learning approach and social immersion through peer to peer interaction. Although this approach does not follow the experience economy framework, it offers valuable insights into creating immersive educational experiences involving active participation that could offer new approaches to educate students on sustainable practices and decision-making. A study investigating cultural immersion to promote Mexican American nutrition and food traditions confirmed these findings [30]. Based on a week-long cultural immersion experience to Mexico, students had the opportunity to immerse themselves in the foreign culture and use participant observation and field visits to develop cultural awareness and understanding related to local food and cooking methods. Using systems immersion, social immersion, and spatial immersion in their educational method by going on the study trip sparked post-experience behavior such as initiatives by students and educators to support the Mexican community. As illustrated in their study, these types of immersive experiences provide promising avenues for intercultural understanding leading to sustainable behavioral change that contribute to societal development. Spatial immersion through VR was also used to educate adolescents about food portion-size in Virtual Cafeterias [33]. In their study, it was revealed that VR could be used to create a learning environment that can provide multisensory feedback and high levels of interaction. Similar to non-technology immersive approaches, emphasis was put on the immersive hands-on learning experience that can be further stimulated through VR. The potential of VR to replicate real conditions was further highlighted in a study on VR buffets [34]. The study revealed that immersive VR environments were a suitable tool to replicate real environments in a controlled research setting that closely mirrored people’s behavior in making food-related decisions. Using VR thus embodies an interesting opportunity to recreate and test real scenarios that can be cost effective solutions that support the triple bottom line of sustainability. Table 1 provides an overview of immersion studies in the food and dining context. It highlights the key findings to date and indicates how the study approaches relate to the experience economy framework. 

The present study extends our current knowledge on mixed reality studies in the food and beverage context and uses Microsoft HoloLens 2 mixed reality device to test the effect of integrated immersive experience facilitators on the consumer experience.

## 3. Research Design

### 3.1. Stimulus Material

**Figure 2:**
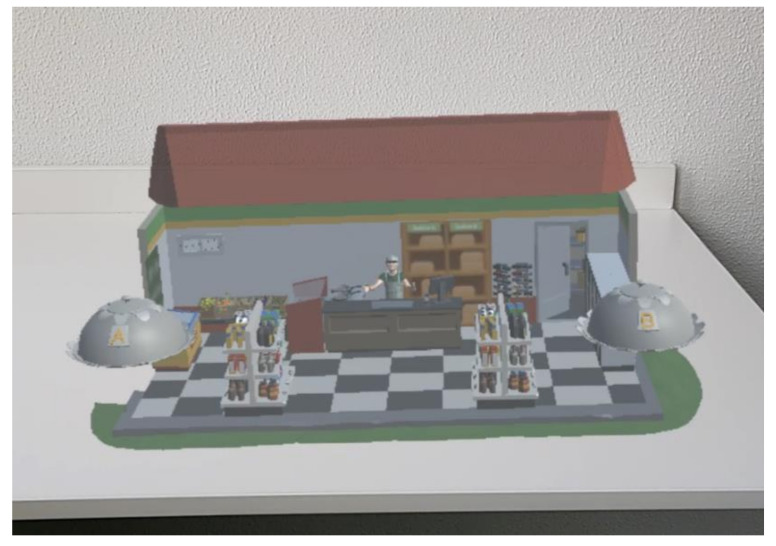
Sample impression Act 1.

**Figure 3:**
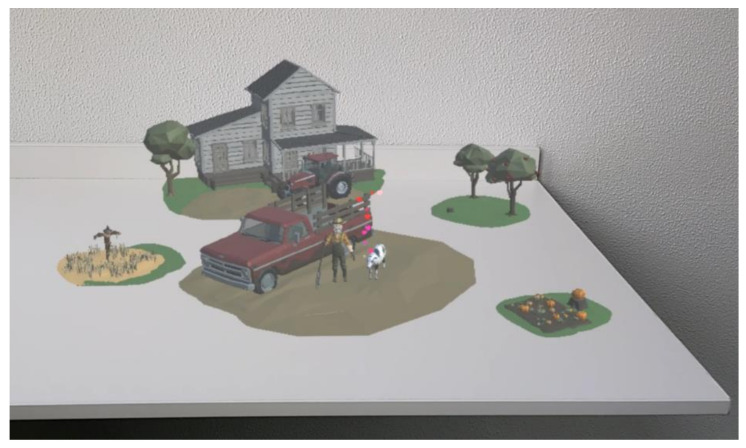
Sample impression Act 2.

A mixed reality interactive content narrative was developed in Unity for the Microsoft HoloLens 2 that consists of a narrative including two acts. The context of the narrative was developed based on young adults (Gen Z) as the target audience for the study. Before the narrative was developed, a sample of potential participants was consulted to discuss the context that would be most relatable and relevant to the target audience. It was concluded that participants were very familiar with a supermarket and farm as contexts for the scenes rather than choosing a restaurant to reflect a dining setting which some participants argued to be less frequently visited. To construct the 3D environment, POLYGON nature pack, town pack, and farm pack were used as the basis for the visual artefacts. The narrative lasted on average 5 min depending on the speed of interaction per participant. The scene revolves around a supermarket (Act 1) and a farm (Act 2) in which the participant is engaging with the scene through characters. Within the narrative, the participant interacts with the content by being prompted to make a choice for a food item or for interacting with characters of the narrative (see Figure 2 and Figure 3 for prototype impression). 

### 3.2. Participants

Purposive sampling was applied to select 20 participants from a Dutch institute. The sample consisted of 6 male and 14 female students enrolled in undergraduate hospitality programs. All participants were fluent in English and gave written informed consent of their participation before the study. Table 2 provides an overview of study participants. The participants were selected on both their affinity with hospitality and their familiarity and skills with modern technology. As this study is based on testing an interactive application in a mixed reality device, it was considered imperative to reduce potential disruption that could be caused by difficulties interacting with the technology. As the majority of participants belong to generation Z, the generation’s skills, familiarity, and experience with modern technology was expected to minimize the probability of the experience being disrupted by issues with the MR technology interaction [35]. In addition, as the study is developed for the context of immersive dining experiences, participants’ affinity with dining and restaurants was considered favorable. As the selected participants are all students enrolled in hospitality programs, they do not only possess this affinity and knowledge of the industry, but also represent the upcoming market of diners who could potentially be confronted with immersive dining experiences provided through mixed reality in the near future. 

### 3.3. Research Design

To accurately measure the impact of the HoloLens content narrative on the participants’ emotions in order to determine whether certain types of immersive experience facilitators were achieved, biometric measures (galvanic skin response) were triangulated with the qualitative self-reported data from the retrospective think aloud protocol.

#### 3.3.1. Retrospective Think Aloud Protocol

To test the immersive effect of the MR content narrative, we applied a “Retrospective Think Aloud Protocol” (RTAP). RTAP is considered a form of Verbal Protocol Analysis in which participants verbalize thoughts and emotions brought on by the content only after having carried out the experience in silence. This is in contrast to the traditional “concurrent-think-aloud method” [36] in which participants verbalize thoughts concurrent with the activities at hand. RTAP was favored over concurrent-think-aloud, as having participants verbalize their thoughts and emotions while experiencing the narrative content might be disruptive to their experience. Furthermore, participants might find it difficult to accurately articulate their experiences whilst also having to engage with the HoloLens 2 content [37]. Retrospective verbal reporting allows participants to be reflective of their choices and thoughts during the process of using the device, which in turn allows them to point out higher-level causes for individual usability problems. Finally, our participants included international students reporting in a language that was not their mother tongue. Retrospective thinking aloud reporting provides a preferable alternative to concurrent think-aloud reporting, as it appears to be less difficult for participants to verbalize their thoughts in a foreign language after the task performance than while actively engaging in the task [36].

#### 3.3.2. Galvanic Skin Response (GSR)

Galvanic skin response (GSR) measurements indicate participants’ arousal triggered by an emotional response to the content narrative by measuring the degree of moisture on the skin released by the sympathetic nervous system. During our study, GSR data were captured using Empatica E4 wristbands which have been shown to be able to record scientifically reliable physiological signals including skin conductance, heart rate and skin temperature [38]. Detailed GSR measurements can be taken continuously and discretely without disrupting the experience or distracting the participant [39]. 

#### 3.3.3. Benefit of RTAP and GSR Data Triangulation

While RTAP offers insight into the participants’ mental processes and immersion effects during the HoloLens 2 content narrative, it lacks objectivity and timeliness [37]. Unbiased, quantitative, detailed, and objective data are effectively captured by biometric measures such as the GSR measured by the Empatica E4 wristbands. However, as GSR data measures effects of heightened emotional response, a higher reading in electrodermal activity could signal both a negative emotional response (e.g., anxiety) or a positive emotional response (e.g., enjoyment), making the data less valuable without context for interpretation.

Relying solely on qualitative self-reported data or biometric data would not allow to accurately capture participants’ emotional responses to the content narrative. Therefore, shortcomings of each method of data collection can be diminished by triangulating the quantitative biometric data of the GSR with the qualitative self-reported data of the RTAP. 

When applying the Verbal Protocol Analysis, we followed the steps outlined in previous studies [40,41]. Having already completed the first step of “devising a scenario” or creating a stimulus material with the HoloLens 2 simulation (see Section 3.1), we proceeded with selecting and training the participants how to “think aloud”. Participants were invited into a controlled environment where the study and procedure were introduced and the Empatica E4 wristbands were attached to the participant. To collect physiological data, Empatica E4 wristbands were used which have been shown to be able to record scientifically reliable physiological signals including skin conductance, heart rate, and skin temperature [38]. A separate video recording captured the entire session, while participants were invited to go through the entire HoloLens 2 interactive content narrative. Their point of view of the entire experience was captured through the recording function in the HoloLens 2. Measurement of physiological responses started a few minutes before engaging with the mixed reality simulation and ended when the interactive content narrative was finished. Once the participants had completed the entire simulation independently, the HoloLens 2 recording was replayed, and participants were prompted to verbalize their thoughts and feelings retrospectively. During this phase, the researcher probed the participant encouraging them to continue talking or ask clarifying follow-up questions. At the end of the experiment, the participant’s Retrospective Think Aloud verbal reports were collected, transcribed, and encoded into categories. Skin conductance data were continuously sampled at a built-in frequency of 4 Hz throughout the session and automatically stored on the wristband for further offline processing. The collected physiological recordings varied between 6.1 min to 7.45 min depending on the speed of participant interaction with the narrative. Finally, the structure, similarities, and frequency of the encodings were analyzed in the data analysis phase. 

### 3.4. Data Analysis

To ensure reliable and verifiable verbal reports that offer insight into the participants’ mental processes and immersion during the HoloLens 2 simulation, we chose to conduct a Verbal Protocol Analysis [42]. A deductive approach was adopted based on Haggis-Burridge’s framework [17] to test the effect of each of the four immersive experience facilitators. 

**Figure 4:**
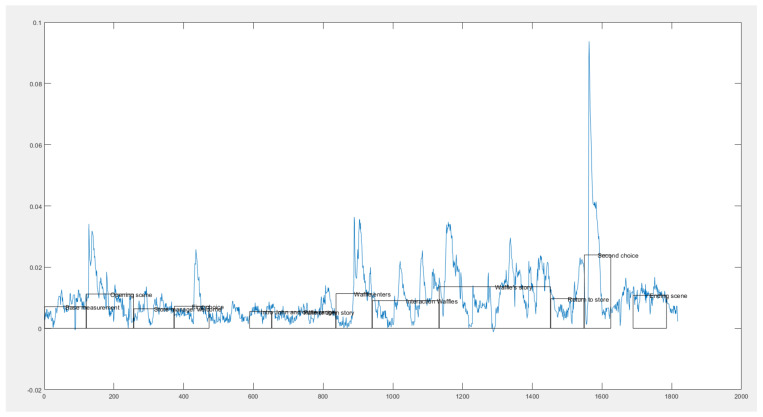
Illustration of the GSR data output.

The mixed reality narrative was segmented a priori into distinctive scenes to facilitate the analysis of physiological data. At the start and end of the immersive experience, a timestamp was recorded to capture the duration of the entire experience, which was then time-synchronized with the HoloLens 2 recording to align the physiological recordings with the on- and offsets of each scene. The data were extracted from the wristbands and imported into MATLAB for further analysis. Processing and analysis of data were performed using a set of MATLAB functions developed at the Experience Lab of Breda University of Applied Sciences that is available in open source on request. As skin conductance data can include motion artifacts from movement of pressure on the recording device [43], an artifact correction was performed to exclude detected peaks that do not correspond to typical GSR amplitudes [44]. Typically, between 3 and 8 artifacts were removed per participant following this procedure. Each participant’s GSR data were subjected to continuous deconvolution using the Ledalab toolbox in MATLAB [45] to split the signal into a tonic and phasic driver. The phasic component consists of a superposition of GSRs which can be closely related to emotional engagement. The output was visualized in a graph (see Figure 4 for example) to allow for data comparison with the qualitative findings from the RTAP.

## 4. Findings

The mixed reality interactive content was divided into thirteen scenes that provided the structure for the coding scheme. For each of the thirteen scenes, the verbal protocols were allocated to the corresponding immersive experience facilitator. For example, in the first scene “opening scene” the protocol “The surroundings, it’s the green light supermarket, and it’s really a small town. I guess this reminds me of my own village.” P16, was allocated to *Spatial Immersion* and coded with “Personal context environment” as the spatial environment presented in the mixed reality content reminded the participant of their personal context. Table 3 presents an overview of codes per respective immersive experience facilitator and the frequency of codes grouped under each facilitator. It reveals that although *Empathic/Social immersion* is not achieved in every scene, most codes and protocols are attributed to it. The most common code within *Empathic/Social immersion* was identified as “Empathy through character narrative” which refers to the participant expressing empathy for the characters through their interpretation of the narrative. For example: *“He sounds like he has good intentions with his farm. I don’t always associate cattle farm with good care for the animals. Like he did care for the animals and they took good care of them” P4.*

### 4.1. Immersive Experience Facilitators across the Mixed Reality Experience

From the results of the Verbal Protocol analysis, it was evident that immersive experience facilitators were mostly occurring simultaneously and not perceived in complete isolation. In Table 4, we depict example quotes from the protocols relating to each immersive experience facilitator.

**Figure 5:**
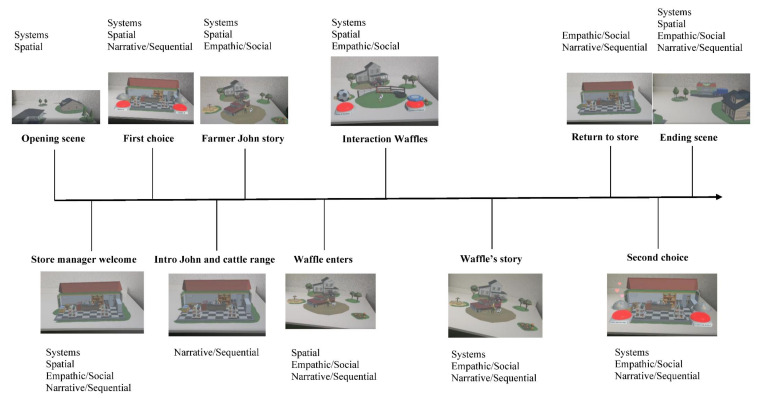
Immersive experience facilitators across the interactive content narrative.

Figure 5 presents a chronological overview of scenes and immersive experience facilitators that were referred to by participants. Through this process, we noted which scenes activated specific types of immersive experience facilitators (see Figure 5). This figure illustrates for instance that the Narrative/Sequential Immersion was experienced by the participants in the majority of the scenes.

### 4.2. Redefining Empathic/Social Immersion

From the protocols, it was evident that the definition of Empathic/Social immersion, as previously conceptualized [17] seemed to be incomplete and needs to be extended in two dimensions. First, while prior research defined Empathic/Social Immersion as a sense of empathy with characters of a virtual environment [17], participants additionally highlighted the empathy that is built with the virtual environment, as participants could relate to esthetic designs of the virtual environment reminding them of their own hometown or personal context. Thus, participants frequently expressed the feeling of emotions in this process.

*“When we entered the supermarket, I thought about this farm shop across from my parent’s house, which is at a farm, and they sell asparagus and strawberries and stuff like that.” (P20)*

Second, according to the protocols, empathic/social immersion seems to be two-dimensional. Participants not only referred to the generating of empathy with characters, illustrating a directive from user to experience, but also expressed empathy that is generated from the environment to self, indicating the second directive of empathy creation while engaging in the interactive content narrative.

*“And it’s like a situation everybody’s been in, at least like, I grew up in the country. Like, you’ve been on a farm, you’ve been to a store like this. The guy behind the counter, I know a guy like that quite well, so it was really easy to visualize yourself in this situation”(P8)*

The protocols further revealed that the design choice of humanizing characters in narratives, following methods of game design, created a stronger perceived bond and empathy for participants. Choosing character design choices that correspond with human characteristics or personality traits (e.g., calf jumping of happiness showing hearts) is an aspect frequently used in games and entertainment to emotionally engage the player or audience in the story [46].

*“And just the hearts coming off the cows head, I think that’s really good. So I know that the cows enjoying it in this simulation, whereas ‘m not just throwing something at him and he’s not reacting.” (P2)*

*“I really like the name Waffles. I kind of focused on the fact that he said he will look forward to it. Because he used ‘he’ and therefore humanized the calf. Instead of ‘it’.” (P5)*

### 4.3. Consumer Choices

The most frequent justification for the food choices (Option A, Option B) made in both Acts of the interactive content narrative was argued to be the perceived novelty of the choice. For example, *“So now, I was really curious about the newcomer items. I’m just curious about new products to taste them. And I said, let’s choose for the new one.” (P14).* In the narrative it was evident that the second choice, while not explicitly mentioning what it was, seemed to be the more interesting option, since the alternative was either the more “common” or “traditional” item as described in the narrative. While this was the case for most participants, the relatively younger participant profile in this study could be an influential factor in making this choice. 

In the second choice, it was evident that participants frequently interpreted the presented choices as beef versus plant-based choice, although this was not explicitly mentioned or written in the content. For instance, “*And so with this, I selected dish A because I was like, I want to do something else, and not the traditional beef version and I do that with my own life as well. I try to make sure that I don’t take too much beef or anything in my diet and try new other things and see if I can change my diet.” (P6)*

This illustrates that consumer perceptions can be framed through storylines to temporarily attain certain consumer mindsets in which assumptions can be inferred. The framing effect in psychology literature, first coined in prospect theory [47], was revealed to be particularly influential in creating cognitive biases to influence people’s choices from a set of options through the way information is presented. This could be particularly beneficial for stimulating sustainable consumer behavior to influence more sustainable consumer food choices through interactive content narratives. 

### 4.4. Embedding Curiosity in Content Design

While this was not intentionally designed in the interactive content narrative used in this study, most participants referred to feeling a sense of curiosity that was triggered by the environment (opening scene) as well as by the development of the narrative. In addition to attempting to generate sequential immersion through the narrative, environment design seemed to be highly effective in drawing participants into the experience.

*“So you’re sort of wondering, but what would it be? I see veggies, I see bottles and different things. So what has he made instead?”* (P15)

An important realization emerged that sufficient transition time into the experience was needed in order for participants to familiarize themselves with the environment and experience a sense of immersion before conscious decisions could be made. P16 for example mentioned, *“Oh, well, uh, yeah, I choose option A because, you know, I’m sorry, but I didn’t even really know what he told me. It was more like, ‘Oh, I have to choose option A.’ First I had to choose Act 1. So I went ‘Ok option A, let’s go!’ Let’s go to the cattle. He already talked like it was that way”.* This phenomenon was also expressed by P20, indicating *“And it was also thinking like, did I miss what he said or did he literally just say newcomer or was there a word I missed? I was mostly wondering that at that moment”.* It was evident that participants struggled particularly with making the first choice, as many were still processing and orientating themselves in the virtual environment, thus, paying little attention to the narrative itself. As a result, several participants noted that they had no confidence in the first choice that they made *“I was going to go with option A and then I thought, that’s basic. I’m going to go with B instead. That was really my only train of thought because at that point I had even forgotten which option was which, so I didn’t actually hear that part of it.” (P9)*

Interestingly, familiarization prior to an immersive experience in mixed reality occurs in two aspects, the familiarization with the virtual environment, much relating to the content familiarization, and second, the familiarization with the technology. Although all participants were selected based on their affinity with consumer technologies, many had never used the Microsoft HoloLens 2 prior to this study. As a result, the novelty of technology seemed to have an influence on their evaluation of the overall experience.

*“It was actually the first time I use glasses like this. It was a new experience, but I actually liked it.” (P7)*

### 4.5. The Role of Technology in Immersive Experiences

While current immersive technologies are increasingly introduced in various contexts in the consumer market, the findings of this study indicated that participants were still affected by the novelty effect of the Microsoft HoloLens 2. Most participants were first-time users of this device and reacted positively to the interaction and technological capacity of augmenting digital content in their immediate environment. According to P9, the perceived novelty of the mixed reality device made a positive contribution to the overall evaluation of the experience, stating *“Kind of felt like the end of a movie that’s set in like the suburbs. I just thought it was really well made. Like I said, I have never done anything like this. So I thought that was really cool.”* However, it was argued that the logical embedding of interactive elements from the mixed reality device into the narrative would further elevate user engagement with the interactive content narrative.

In alignment with previous study findings [48], the outcomes of this study confirm that a key threshold to be addressed is the smooth and glitch-free interaction with the technology to allow users to completely focus on the content and intended experience. It was highlighted that interacting with mixed reality required the sense of full control in the interaction with digital content to be able to remain in the immersive experience.

*“That it was too much is just your first impression because you just you don’t know how the glasses work. I have never seen it before. So then the first Act is really too much. So when the first person, the owner of the shop, talked to you I was just more focused on. ‘Oh, my God, there is a shop. I see it in front of me. But now you’re getting used to. Okay, so I was talking to you. You have a scene and you can choose the options. Yeah. You’re just getting more used to it. So then it’s not too much.”(P16)*

### 4.6. Narrative Design

During the RTAP, participants often discussed perceived unrealistic aspects to the interaction. Instances where the coherency of the narrative was broken were for example, a cow playing with a frisbee, as noted by P10, *“This again was really weird. Cows probably not play with things like that”,* or even the unrealistically perceived act of playing with a calf, as mentioned by P15: *“There I had to play with the cow, which I thought was really strange, because you don’t play with cows. You just leave them be”.* As these aspects of the interaction seem to be distracting the participants, it could be argued that these participants are taken out of immersion as a result.

In the scene where the participant interacts with Waffles the cow, we observed a general tendency in all participants for higher readings in their skin conductance data, indicating a higher emotional response because of the experience (see Figure 4 for illustration). These spikes could be interpreted as the participant experiencing happy emotions of bonding, as indicated by P6, *“When seeing the hearts it felt like a nice experience like I was sort connected to it.”* Interestingly, the possible disturbing aspect of the perceived unrealistic interaction with Waffles might have caused negative emotions due to the participant’s feelings of confusion, providing an alternative explanation for some participants’ higher skin conductance.

Although the perceived incoherency in the narrative might have caused negative emotional spikes during the scene where the participants interact with the cow, our RTAP qualitative data show that many participants felt positive emotions in the subsequent scene, where Farmer John reflects on the visit and remarks how happy the participant has made Waffles the cow (see Figure 4 for illustration). From the RTAP data, it could be interpreted that reflecting on the interaction that originally may have led to negative emotions in the moment, and framing it in a positive way through the narration, seems to have created positive emotion-induced spikes in skin conductance. To illustrate, P4, who initially remarked *“I was thinking, what would a cow do with a Frisbee? I don’t know. Probably not much.”* for the interactive scene, commented in the following scene, *“This made me feel happy. Even though it’s just virtual. I also felt positive about the farmer because he cared for the calf.”*

The narrative design seemed to further trigger significant reactions in participants’ reflection on their own (un)sustainable habits and other sustainability issues due to the script spoken by the character Farmer John. The scene in which Famer John talks about Waffle’s sister and sustainable nutrition of his cattle, prompted many participants to discuss their thoughts and feelings on sustainability within the food system, such as P14, “*At this moment, I was also thinking about chicken because a lot of times chickens are put in in a small cave in size and sometimes you have the choice for different egg types for like free-range chickens. I was thinking about cows at those moments. How if milk could be also, like how you could choose for a happy cow milk.”* or the sustainability of their own consumption habits, such as P18, *“I already cook more vegetarian but I like the structure of meat. So I choose a lot of times just other options like vegetarian meat, instead of no meat at all. But sometimes I do eat meat. When I go to a tapas bar or restaurants but now when I go to a restaurant and he shows me this, I don’t know, I will never choose traditional products I will choose the cow.”*

## 5. Discussion

The positive and engaged response from the participants in the study indicates that there is both room and desire for innovation in the experience economy, and that technology can play a role in this. Our study findings suggest that immersion can play a key role in enhancing the impact of the experience for educational as well as entertaining experiences mediated by immersive technology. Multiple senses can be engaged in immersive experiences as a mechanism to detach consumers from the immediate physical surrounding into an alternative reality. In contrast to how the experience economy framework [16] is depicted, our study indicates that creating an immersive experience through mixed reality can contribute to entertainment experiences through active participation in an interactive content narrative. Furthermore, immersion does not only seem to be dominant in aesthetic and escapist experiences, but can also play a key role in stimulating entertainment and learning experiences. The outcomes of this study revealed that multiple immersive experience facilitators occur simultaneously and that interactive content narratives can be used to enhance the effectiveness of one facilitator, e.g., social/narrative immersion over others. Perceived novelty and curiosity were identified as dominant motivators to contribute to immersive experiences in stimulating consumers to follow the unfolding of narratives as well as steering of consumer choices. This is particularly interesting for the simulation of choices that can address a more sustainable pathway for consumers, as such choices can be repackaged into scenarios that trigger curiosity rather than educating on well-known sustainability issues. This means that consumer behavior in the food and dining context can be stimulated through sparking curiosity in the design of food products through presentation, engaging narratives, and careful choices of artifacts in the environment setup. Consumers in controlled situations seem to be willing to choose beyond what is familiar and expected, and less prone to take risks in making food choices that can provide new food experiences. However, the study revealed that the sole use of novel technology such as mixed or augmented reality for instance for the presentation of menu items to stimulate consumer choices [49] will not suffice to engage consumers through activating curiosity and interest. While perceived novelty in content design through novel technologies needs to be considered with care as the novelty factor is typically expected to diminish after repeated use, interactive content narratives offer a promising approach to alternate storylines and developments of narratives.

Our findings revealed that the participants expressed empathy for the characters in the narrative and that the design choice of humanizing characters created a stronger perceived bond and enhanced the level of empathy toward them. This is in line with findings of previous studies [8], where guests experiencing an immersive experience in the context of Eat & Travel built meaningful connections with the NPC (Non-Player Character) captain Jones. By learning about a destination’s culture and cuisine through the passionate and personal thoughts of the Captain’s journal, the NPC was “humanized”. Similarly, a study on the immersive international learning study experience [30], demonstrated that the included interactions with the citizens of Guanajuato Mexico improved students’ understanding of the dimensions involved in caring for people from culturally diverse backgrounds. In the same way that connection and empathy toward the NPC “Captain Jones” or the community members of Guanajuato could facilitate and enhance the learning about the local culture and cuisine and enhance care and understanding, empathizing with Farmer John or Waffles the calf in the context of the narrative of this study could allow for a deeper absorption of knowledge and understanding of the messages presented in the narrative. This finding demonstrates that MR could be a suitable technology to address evolutionary tendencies [6] that make it challenging for people to act sustainably. For instance, bringing extant scenarios of societal issues in other parts of the world that are caused through damaging the environment into the direct vicinity of consumers could help consumers to build empathy and better grasp the impact of many of our current unsustainable behaviors, addressing our propensity for self-interest as well as a predisposition to be short-sighted and our proneness to disregard impalpable concerns. Similarly, creating MR content that enables consumers to compare themselves and their own behavior and performance to others could trigger actions toward sustainable behaviors that are rooted in our motivation for relative rather than absolute status and proclivity to unconsciously copy others, thus exploiting two of the evolutionary tendencies [6]. 

From a methodological perspective, the findings of the study suggest that use of galvanic skin response measurements in combination with qualitative research is a suitable approach to confirm and interpret physiological readings in consumer research. As higher readings in electrodermal activity can suggest a positive as well as negative emotional response, qualitative research to underpin the measurements could be more appropriate than the combination of self-report questionnaires in combination with physiological measurements as commonly performed in prior studies. As a research approach, this study has shown that consumers can emotionally engage with MR content even while in a dining situation. The correlations between the content of the media and the galvanic skin response readings, implying that participants were having fun and feeling attachment to the virtual characters, show that Empathic/Social immersive connections are possible in a testing situation. This first result suggests that the methodology is sound enough to examine further digital experiences in this manner to enhance our understanding of immersive experiences in the dining context.

## 6. Conclusions

The present study tested the immersive experience of an interactive content narrative developed for Microsoft HoloLens 2 mixed reality glasses with the aim of extending our understanding of the experience economy framework [16]. Theoretically, this study expands our current understanding of immersive experiences and their design criteria. Although the limitations of our study through involving participants based in the Netherlands only need to be acknowledged, the study makes an attempt to unpack the realm of “immersion” in the experience economy framework [16]. It sheds light on how immersive experiences can be generated through interactive content narratives in mixed reality, thus adding to the current body of knowledge on the effects of immersive environments [14]. This study adds an additional methodological approach to recent studies adopting physiological measurements to examine food consumption experiences [50]. These complementing methodologies that provide opportunities for data triangulation offer valuable insights into internal consumer responses (e.g., emotions) that have profound effects on resulting consumer attitude and behavior. Our findings also hold practical implications for professionals that are working with immersive experiences. The study in particular makes a case for immersive experiences using MR to stimulate consumer choices through interactive content narratives. Thus, experiences in the food consumption context can be repackaged with the aim of reinterpreting the meaning of choices through digital content in the immediate physical environment of consumers. While the adoption of MR by restaurants to date is limited, generating immersive experiences in MR that can spark curiosity in consumers offers practitioners a valuable tool to stimulate consumer choices of specific food items. This approach also provides an opportunity to better integrate their brand with cutting edge technology in interactive content narratives that are appealing to consumers. 

While there is much to be learned from upcoming hardware and software developments in this field, the combination of using interactive content narratives in mixed reality offers promising avenues for future research to expand on the findings of this study. The experience economy framework [16] provides a foundational understanding of types of experiences that can be triggered. However, we advocate that further research is needed to explore the underlying mechanisms of such experiences. In the lens of designing experiences that are transformative, future research could focus on expanding our understanding on criteria that facilitate this transformation. A better understanding how these experiences unfold through integration of emerging technologies could grant us with a clearer pathway toward designing for transformative experiences that can benefit consumers, the community, and the environment through more sustainable choices. 

## Figures and Tables

**Table 1:** Overview of immersion studies in the food and dining context.

Author	Study	Type of Immersion	Sample and Method	Key Findings	Related Realms of the Experience Economy
10. Dionísio et al. (2013)	Testing Eat & Travel prototype solution in immersive CAVE setting	Spatial Social Narrative	Proof of concept	Immersive CAVE setup engages guests to interact and explore. Guests perceived to be immersed and sense of presence in the virtual environment. Guests have the desire to learn and share information about the food and destination. Meaningful connection can be established between guest and NPC (non-player character)	Escapist Entertainment
35. Qvist et al. (2016)	Elevating the level of immersion in CAVE environments for location independent multisensory gastronomic services	Spatial	Proof of concept	Multisensory CAVE settings can be used to provide sensory and cognitive engagement in form of an immersive dining experience.	Escapist
38. Sester et al. (2012)	Evaluate the effect of contextual stimuli through visual projections on drink choices	Spatial	Experimental study in two immersive bars (Study 1: N = 176; Study 2: N = 120)	Food behavior can be tested through immersive CAVE setups to test various contextual influences on food behavior	Escapist Esthetic
46. Ung et al. (2018)	Use of VR to examine people’s real food choices	Spatial	Experimental study (N = 34)	VR is a suitable tool for researching people’s real food choice behavior	Escapist
30. Perez et al. (2019)	Distributed Reality as innovative concept for creating immersive gastronomic experiences	Spatial	System evaluation study (N = 66)	Distributed Reality provides an innovative concept in hospitality and tourism to combine local and remote environments through VR	Escapist
18. Harper et al. (2003)	Immersion approach to teach basic concepts of food science	Systems Social	Analysis of pre- and post-test results (N = 22)	Immersive, team-based teaching approach helps students recall basic concepts and acquire a broad knowledge base in food science	Educational
13. Gilboy & Bill (2011)	Cultural immersion experience as educational method to increase cultural awareness and understanding	Systems Empathic Spatial	Content analysis on reflective participant journals (N = 16)	Cultural immersive experiences increase understanding of foreign cultures and can promote behavioral change post-experience	Escapist Educational
4. Celikcan et al. (2018)	Immersive virtual environment for interactive food portion-size education	Spatial Systems	Usability test of the virtual environment	High detailed virtual environments through VR can be used for immersive educational purposes providing multisensory immediate feedback and high level of interaction	Escapist Educational
36. Ranasinghe et al. (2016)	Use of electrical pulses and virtual overlays to create and modify Immersive taste experiences	Systems	Experimental study in laboratory setting	Technological modifications (virtual food and beverage ingredients) are able to change taste experiences in food and beverage consumption and generate immersive experiences	Esthetic

**Table 2:** Participant profiles.

Participant Code	Gender (M/F)	Country of Origin
P1	F	The Netherlands
P2	F	Taiwan
P3	F	The Netherlands
P4	M	The Netherlands
P5	M	Germany
P6	M	The Netherlands
P7	F	The Netherlands
P8	M	The Netherlands
P9	F	India
P10	M	Germany
P11	F	The Netherlands
P12	F	The Netherlands
P13	M	Syria
P14	F	The Netherlands
P15	F	The Netherlands
P16	F	The Netherlands
P17	F	The Netherlands
P18	F	The Netherlands
P19	F	The Netherlands
P20	F	The Netherlands

**Table 3:** Codes corresponding to immersive experience facilitators.

	Systems Immersion	Spatial Immersion	Empathic/Social Immersion	Narrative/Sequential Immersion
**Codes**	MR user experience	Personal context environment	Empathy through character narrative	Curiosity
	User Interaction (UI)	VE impression	Empathy through character narrative	Curiosity
	Distraction through device interaction	Sustainability in VE	Personal context environment	Choice based on novelty
	Empathy through character interaction	Intentional behavior through VE	Empathy through character narrative	Bewilderment of choice
	VE impression	VE impression	Reflection on sustainability issues	Distraction through content narrative
	MR user experience	choice based on personal context	Personal context environment	Personal context environment
	MR user experience	Reflection on sustainability issues	Distraction through content narrative	Distraction through content narrative
	MR user experience	Exclusive shopping experience	Intentional behavior through VE	Rational processing of narrative
	MR user experience	Personal context environment	Intentional behavior through VE	Sustainability in narrative
		VE impression	Distraction through content narrative	Rational processing of narrative
		VE impression	Personal context narrative	Rational processing of narrative
		MR User experience	Empathy through character interaction	Curiosity
		choice based on personal context	Reflection through narrative	Reflection on sustainability issues
		Experience evaluation	Intentional behavior through VE	Rational processing of narrative
		VE impression	Empathy through character interaction	Curiosity
			Distraction through content narrative	Distraction through content narrative
			Personal context narrative	Choice based on novelty
			Empathy through character narrative	choice based on personal context
			Intentional behavior through VE	Bewilderment of choice
			Personal context narrative	Interpretation of objects
			Reflection on sustainability issues	Curiosity
			Experience evaluation	Rational processing of narrative
			Reflection through narrative	
			Reflection on sustainability issues	

**Table 4:** Quotes per immersive experience facilitator.

Immersive Experience Facilitator	Example Quote
Systems Immersion	*“It was also really cool to have my first experience with VR. I did not have that already. The view was really clear and the audio also.” P3*
Spatial Immersion	*“It felt very realistic, surprisingly. There was a lot going on, I was looking around.” P4*
Empathic/Social Immersion	*“When he told me that it would make him really happy. I thought, ‘Oh, I would love to do make him even more happy.” P14*
Narrative/Sequential Immersion	*“So here I understood that by letting the cow live outside and more freely without being caged makes them not only happier but also just healthier. And that in turn also affects the food we eat that that the cow produces.” P2*

## Data Availability

All data of this study were handled anonymously and in full compliance with the GDPR and the Netherlands Research Integrity Code. The data is stored for the long term on Microsoft Sharepoint of Breda University of Applied Sciences for further processing and use. Microsoft Sharepoint of Breda University of Applied Sciences is a safe and secure environment supported by the IM/ICT department of the institute.
